# Supplementary material for: Aromatic Molecular Compatibility Attenuates Influenza Virus-Induced Acute Lung Injury via the Lung–Gut Axis and Lipid Droplet Modulation
Source: Pharmaceuticals (Basel). 2025 Mar 26;18(4):468. doi: 10.3390/ph18040468 (PMC12030469; doi:10.3390/ph18040468)
Supplement: Supplementary file 1 [file pharmaceuticals-18-00468-s001.zip › pharmaceuticals-3545897-supplementary.pdf]

Supplementary Materials for

An aromatic molecular compatibility attenuates influenza virus-induced acute lung injury via the lung-gut axis and modulation of host lipid droplets

Yi Li[1], Jiakang Jiao[2], Haoyi Qiao[1], Conghui Wang[1], Linze Li[1], Fengyu Jin[1], Danni Ye[1], Yawen Chen[1], Qi Zhang[1], Min Li[3], Zhongpeng Zhao[4], Jianjun Zhang[2*], Linyuan Wang[1*]

[1] School of Chinese Materia Medica, Beijing University of Chinese Medicine, Beijing, 102488, China.

[2] School of Traditional Chinese Medicine, Beijing University of Chinese Medicine, 102401, Beijing, China.

[3] State Key Laboratory of Pathogen and Biosecurity, Beijing Institute of Microbiology and Epidemiology, AMMS, Beijing, 100071, China.

[4] Beijing Minhai Biotechnology Co., Ltd., Beijing, 102600, China.

This word file includes:

Supplementary Materials and Methods

Figures S1 to S5

Tables S1 to S2

* Corresponding authors:

E-mail addresses: wangly@bucm.edu.cn (Linyuan Wang)

zhangjianjun@bucm.edu.cn (Jianjun Zhang)

Supplementary Materials and Methods

Median Tissue culture infectious dose (TCID50) assay

As previously described, TCID50 assays were conducted with MDCK and A549 cells to ascertain the infectivity of the PR8 strain. Ten-fold serial dilutions of the viral stock solution were prepared in DMEM containing 1 μg/ml TPCK-trypsin. Each 100 μl of the viral stock solution was then transferred to a monolayer of cells grown on 96-well plates and incubated at 37°C in a 5% CO_2 incubator. The growth of the cells was observed and recorded on a daily basis, with 12-hour intervals for cytopathogenic effects (CPE), as evidenced by rounding and shedding of infected cells. Once the development of cytopathic lesions ceased (72 hours in this study), the phenomena of cell shedding and reduction no longer changed. The TCID50 of the virus was calculated according to the Reed-Muench method, which is based on the Standard Operating Procedures for Chinese National Influenza Center.The H1N1 experiments were carried out in a biosafety level 2 (BSL-2) laboratory in accordance with the protocol of the study.

Immunofluorescence assay

Paraffin sections of mouse lung tissue from each group were prepared, deparaffinized, rehydrated with a gradient ethanol solution, and heated with citrate buffer for antigen repair. Subsequently, a primary antibody F4/80 (1:100) was added, incubated in a wet box at 4 °C for 15 h, protected from light, and fluorescent secondary antibody staining (red) was added. Serum was then added to seal the sections, and a primary antibody CD206 (1:200) or iNOS (1: 50) was added to each of the sections, which were incubated at 4 °C for 15 h, protected from light, and fluorescent secondary antibody staining (green) was added to the sections. The nuclei were then stained, the sections were sealed, and the images were observed and captured under a microscope.

The mouse colon tissues were processed as previously described, with the addition of a primary antibody against ZO-1 (1:100). They were incubated at 4°C for 15h, followed by the addition of a fluorescent secondary antibody (green). The tissues

were then incubated at 4°C for a further 15h with a fluorescent secondary antibody (red), after which the primary antibody against Occludin (1:400) was added. After a further incubation period of 15h, the fluorescent secondary antibody staining (red) was applied. The nuclei were restained, the slices blocked, and images captured under a microscope for observation.

The frozen sections were thawed at room temperature and stained according to the instructions provided with the DHE kit. They were then washed and sealed and images were taken under a microscope.

The distribution of LDs in cells was observed using an inverted fluorescence microscope according to the procedure outlined in the Nile Red Staining Kit. Mouse lung tissue sections were incubated with BODIPY 493/50 solution (1:1000) at room temperature for 15 minutes. The nuclei were re-solubilized with DAPI and then sealed, and the images were captured under the microscope.

Supplementary figures

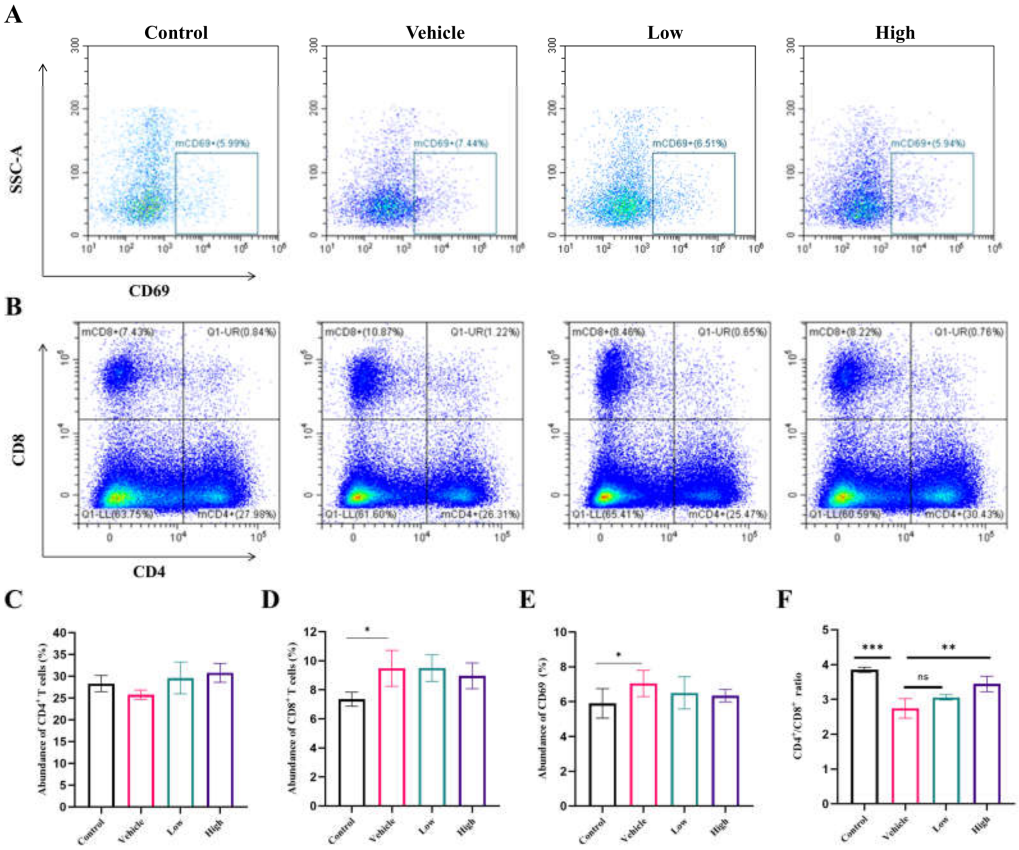

Figure S1. (A-F) The effect of flow cytometric analysis of AC on the expression of $CD4^+$,$CD8^+$ and $CD69^+$ in the spleen of mice with IAV-induced ALI and its quantification. The data are presented as the mean ± SD, n=3 * P < 0.05, ** P < 0.01, and

*** P < 0.001 , ns: not significant.

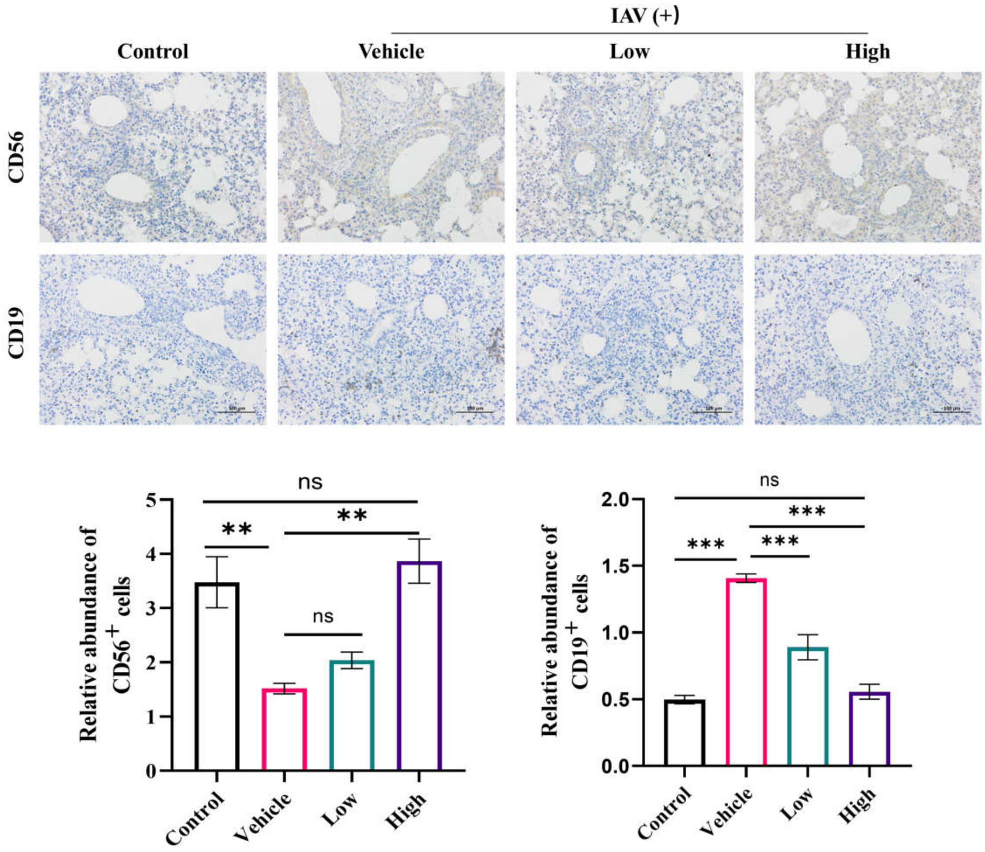

Figure S2. (A) Effect of AC on $CD56^+$,$CD19^+$ expression in lung tissues of mice with IAV-induced ALI and (B, C) quantification. The data are presented as the mean ± SD, n=3. * $P < 0.05$, ** $P < 0.01$, *** $P < 0.001$, ns: not significant.

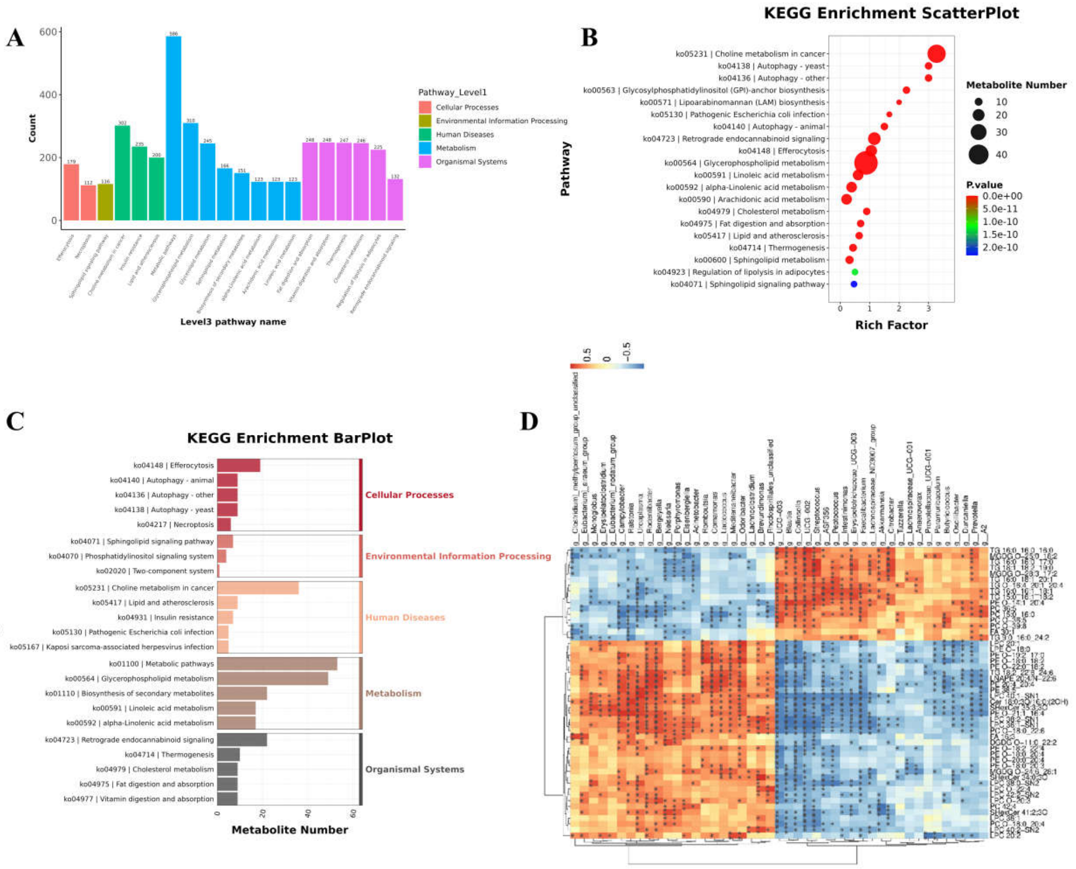

Figure S3. Lipid metabolism effects of AC on serum of IAV-induced ALI mice. (A) Metabolite number TOP20 of the KEGG level3 pathway. (B) KEGG enrichment analysis, (C) Enrichment scatter plot showing changes in serum lipid metabolic processes after IAV infection. (D) The Spearman correlation coefficients between the abundance of the top 45 differentially enriched Enterobacteriaceae genera and the top 45 differentially enriched metabolites. Correlation effects are indicated by a color gradient from red (positive correlation) to blue (negative correlation).

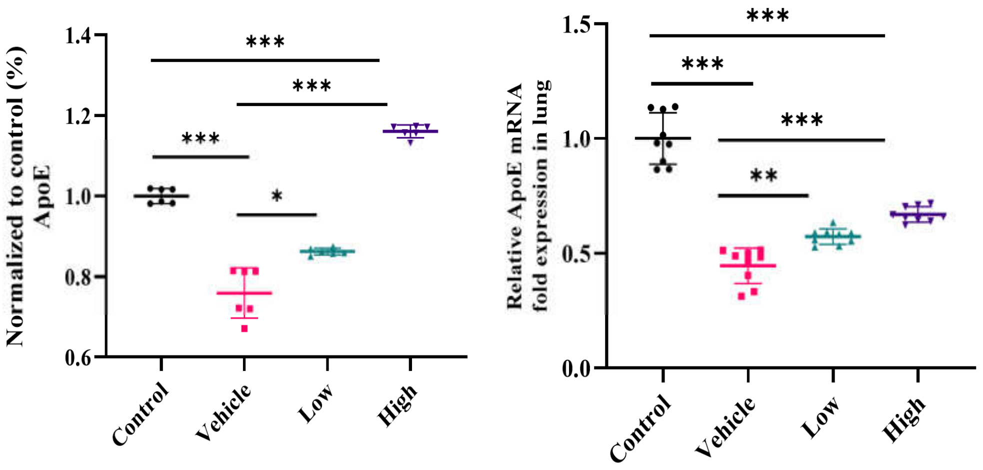

Figure S4. The regulation of ApoE expression by AC in A549 cells (n=6) **as well as in IAV-induced ALI mice** (n=9)**.** The data are presented as the mean ± SD, * $P < 0.05$, ** $P < 0.01$, *** $P < 0.001$, ns: not significant.

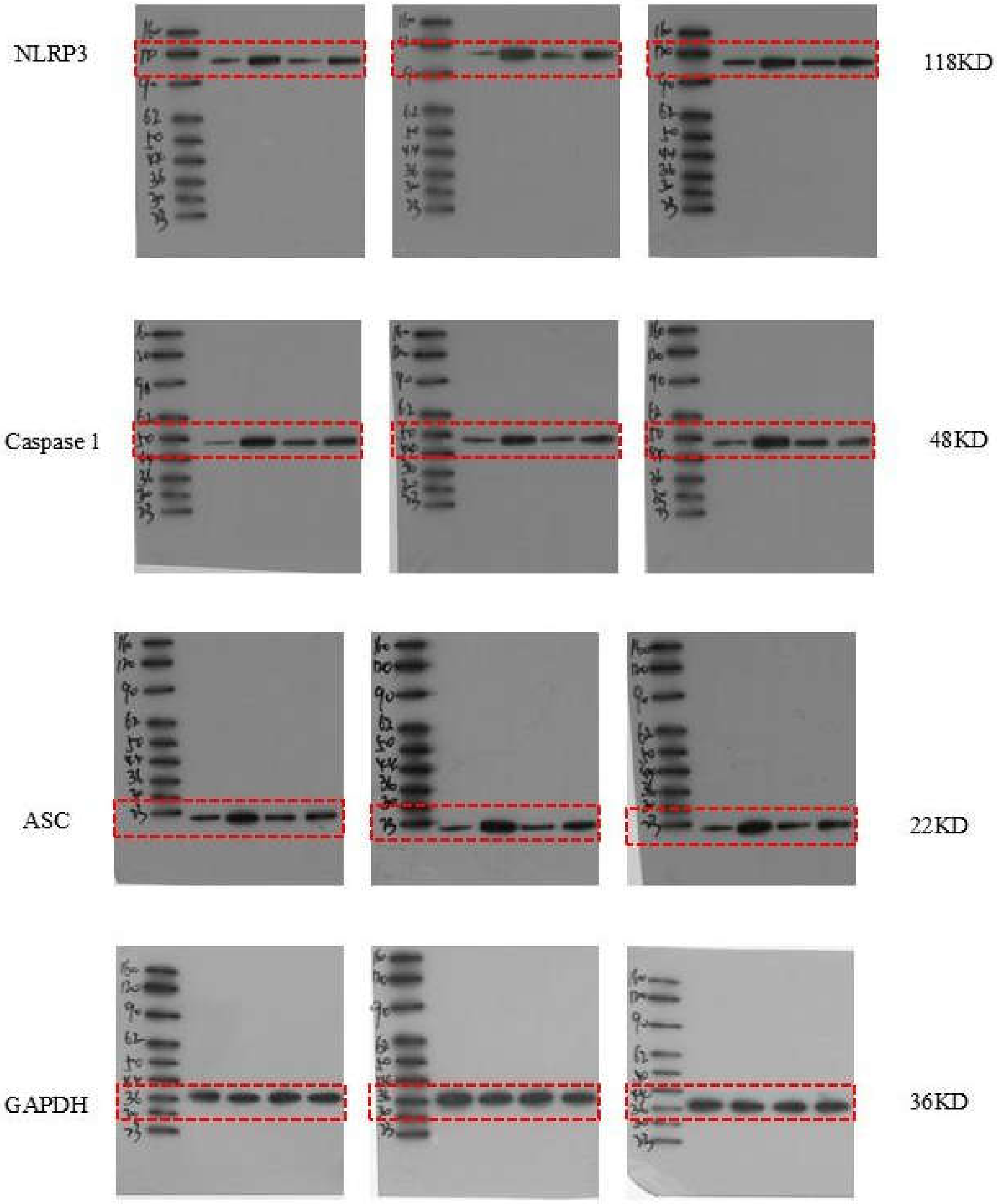

Figure S5. Original protein bands for NLRP3, Caspase1, ASC and GAPDH. (n=3)

Supplementary Tables

Table S1. Sequences of the primers used in this study

Gene	Sequence
IAV-NP	F: 5’-GTCAGAATGATCAAACGTGGGA-3’
	R: 5’-TACGGCAGGTCCATACACACAG-3’
Mouse ApoE	F: 5’-CTGACAGGATGCCTAGCCG-3’
	R: 5’-CGCAGGTAATCCCAGAAGC-3’
GAPDH	F: 5’-TCATTGACCTCAACTACATGG-3’
	R: 5’-TCGCTCCTGGAAGATGGTG-3’

Table S2. The half cytotoxicity concentration (CC50) and half-maximal effective concentration (IC50) of patchouli alcohol, carvacrol, p-cymene, eucalyptol, AC and oseltamivir against different cell lines was determined by CCK8 method. (n=3）

Group	MDCK		A549	
	**CC50 (μg/mL)**	**IC50 (μg/mL)**	**CC50 (μg/mL)**	**IC50 (μg/mL)**
Patchouli alcohol	$34.71 \pm 0.52^{***}$	$7.32 \pm 0.46^{***}$	$36.80 \pm 0.66^{***}$	$5.74 \pm 0.24^{***}$
Carvacrol	$43.31 \pm 6.51^{***}$	$10.35 \pm 1.06^{***}$	$35.80 \pm 3.34^{***}$	$6.74 \pm 1.08^{***}$
p-Cymene	$59.57 \pm 4.57^{***}$	$31.60 \pm 2.18^{***}$	$61.30 \pm 6.64^{***}$	$20.43 \pm 1.88^{***}$
Eucalyptol	$93.15 \pm 5.87^{***}$	14.21 ± 0.52	$86.23 \pm 4.63^{***}$	11.93 ± 0.73
AC	$168.90 \pm 3.09^{***}$	$8.10 \pm 0.50^{***}$	$205.80 \pm 7.03^{**}$	$10.43 \pm 1.0^{**}$
Oseltamivi	711.65 ± 179.82	15.52 ± 1.00	884.40 ± 459.85	14.64 ± 1.36

Compared with the Oseltamivi group, $^{**}P < 0.01$，$^{***}P < 0.001$.
